# Kondo effects in small-bandgap carbon nanotube quantum dots

**DOI:** 10.3762/bjnano.11.169

**Published:** 2020-12-23

**Authors:** Patryk Florków, Damian Krychowski, Stanisław Lipiński

**Affiliations:** 1 Department of Theory of Nanostructures, Institute of Molecular Physics, Polish Academy of Sciences, M. Smoluchowskiego 17,60-179 Poznań, Poland

**Keywords:** carbon nanotubes, Kondo effect, mesoscopic transport, quantum dots, valleytronics

## Abstract

We study the magnetoconductance of small-bandgap carbon nanotube quantum dots in the presence of spin–orbit coupling in the strong-correlations regime. A finite-U slave-boson mean-field approach is used to study many-body effects. Different degeneracies are restored in a magnetic field and Kondo effects of different symmetries arise, including SU(3) effects of different types. Full spin–orbital degeneracy might be recovered at zero field and, correspondingly, the SU(4) Kondo effect sets in. We point out the possibility of the occurrence of electron–hole Kondo effects in slanting magnetic fields, which we predict to occur in magnetic fields with an orientation close to perpendicular. When the field approaches a transverse orientation a crossover from SU(2) or SU(3) symmetry into SU(4) is observed.

## Introduction

Due to their remarkable electronic, transport, mechanical, and magnetic properties carbon nanotubes (CNTs) are of great interest in molecular electronics and spintronics with potential applications, for example, as field-effect transistors, nanoelectromechanical devices, logic gates, spin valves, spin diodes, and spin batteries [1–11]. CNTs are also interesting for fundamental science. Their study allows for the examination of many basic properties in ranges often not reachable in other systems. Many of the fundamental transport properties were observed in nanotubes, including Coulomb blockade [12–13], Fabry–Perot interference [13–14], Kondo physics [15–29], spintronic effects [10,30–32], and induced superconductivity [33–35].

In the present paper, we are interested in the effects of strong correlations. As electrons are confined in fewer dimensions, the effects of interaction play a more fundamental role. Carbon nanotubes are quasi one-dimensional systems, and the role of correlations further increases in quantum dots due to additional confinement. Of importance is also the low dielectric constant, which is especially low in suspended nanotubes [36].

A single-walled carbon nanotube is a hollow cylinder formed of graphene. A CNT can be either metallic or semiconducting, depending on the way graphene is rolled up [37–38]. In the simple “zone folding” picture [39–40], the band structure of CNTs is obtained from the band structure of graphene by imposing periodic conditions along the circumference. When the closest quantization line misses the *K* point, a bandgap appears. The bandgap depends on the minimum separation of the circular quantization lines from the Dirac points. The semiconducting gaps are of the order of a few hundred millielectronvolts [9]. The nanotubes are characterized by the chiral vector *C*(*n*,*m*), where the integers *n* and *m* denote the number of unit vectors along two directions in the honeycomb crystal lattice of graphene. Zone folding theory predicts nanotubes to be metallic if *n* − *m* is a multiple of three. According to this prediction, one third of randomly selected nanotubes should be metallic. Experimentally, the fraction of nanotubes showing metallic behavior is very small (≤1% [41]). Even in nominally metallic tubes a narrow gap of the order of 10 meV is usually observed. These systems are sometimes called “nearly metallic” carbon nanotubes [42–43]. The reason for incorrect predictions of the zone folding theory for small-diameter tubes is the neglect of the curvature-induced breaking of the three-fold (*C*_3_) rotational symmetry. When graphene is deformed into a nanotube, the curvature causes the overlap matrix elements to depend on the direction [44]. The consequence of symmetry breaking is a shift of the Dirac points in the reciprocal lattice away from the *K* and *K*’ points. The broken symmetry also enhances the intrinsic spin–orbit (SO) coupling in carbon nanotubes compared with flat graphene. Apart from curvature [44–47], other perturbations such as axial strain or twists can shift the dispersion cones in CNTs and open the bandgap [44,48–50]. Unlike the quantization bandgaps, which depend on the inverse of the diameter, these narrow perturbation gaps are inversely proportional to the square of the diameter and depend on the chiral angle. Small gaps are reflected in nonlinear dispersion curves and consequent drastic changes of orbital effects induced by magnetic fields. The behavior in magnetic fields is distinctly different than in wide-bandgap nanotubes. The field dependencies are determined not only by the response of orbital and spin magnetic moments, as in the case of large gaps. They also crucially depend on the value of the bandgap and the gate voltage. Details of the band structure are decisive for the response on the field. The degeneracy recovery lines plotted in the plane of magnetic field and gate voltage are no longer parallel to the gate-voltage axis, like in wide-bandgap tubes. Instead, they depend on the gate voltage and they intersect at certain fields with other similar lines, which leads to the appearance of higher degeneracy in the system. The presence of different degeneracy points and the gate dependence of degeneracy lines are interesting for quantum computing because they open the possibility of electric switching between different types of qubits (spin, valley, or valley–spin) and their higher-dimensional equivalents (qutrits [51–52], qudits [53–54]) in the same nanoscopic system. The storage capacity of three-state or four-state qudits is obviously higher than the capacity of a qubit. In the present paper, we are interested in transport properties. The regime of charge transport depends on the ratio between tunnel-induced broadening of dot energy levels and charging energy. For very weak dot–lead coupling and strong Coulomb interaction, the electrons enter the dot one by one and Coulomb-blockade oscillations of conductance are observed. For stronger coupling to electrodes, higher-order tunneling processes (i.e., cotunneling) begin to play a decisive role. Also, many-body resonances form at the Fermi level, which enable transport in the valleys between Coulomb peaks. In CNTs spin–orbit interaction plays an important role. The first experimental evidence showing the significance of this effect was the observation of splitting of a spin–orbital quadruplet into two Kramers doublets recorded by single-electron spectroscopy carried out on ultraclean nanotubes [55]. Later, other experiments confirmed the fact that due to SO coupling spin and orbital degrees of freedom are not independent even in the absence of magnetic fields [42,56–59]. SO coupling is an effect that emerges because of the curvature of the CNTs [60]. Due to the curvature the inversion symmetry is broken and hopping between p-orbitals of different parity from neighboring atoms is possible, which enhances SO coupling. Depending on the sign of SO coupling, this interaction introduces parallel or antiparallel alignments of spin and angular momentum. The energy of SO coupling is comparable to the energy scale of the Kondo effect. Therefore, taking this perturbation into account is important when analyzing many-body effects in these systems. Several interesting papers have been devoted to the problem of interplay of Kondo effect and SO interaction [26–27,29,61–62]. In our previous paper [26], we have pointed out the possibility of the occurrence of the SU(3) Kondo effect in wide-bandgap nanotubes as a consequence of the threefold degeneracy induced by intervalley exchange or due to intershell mixing. We also announced in [26] the possibility of the occurrence of the SU(4) Kondo effect in narrow-bandgap nanotubes despite the presence of SO coupling. In the present paper, we show that the Kondo physics of narrow-bandgap nanotubes is much richer. Apart from SU(2) Kondo resonances with effective spin, valley, or spin–valley fluctuations, the emergence of an exotic SU(3) Kondo resonance is foreseen even without mixing between shells or valleys, but simply due to the peculiarity of the band structure and a subtle interplay of magnetic field, spin–orbit interaction, and changes of the bandgap. In the following we show how, for a given nearly metallic nanotube, one can change the position of high-symmetry points by strain and a magnetic field. Our calculations also show that in a quantum dot formed in a small-bandgap nanotube electron and hole states can degenerate in slanting magnetic fields. Based on this observation we anticipate the possible occurrence of Kondo effects in which both types of carriers take part. Apart from SU(2) Kondo lines, also SU(3) Kondo points and SU(4) may appear for orientations of the field close to perpendicular.

## Model and Formalism

In our analysis we consider the low-energy and low-temperature ranges. Therefore, we restrict most parts of our discussion to only a single shell of carbon nanotube energy states, that is, to four states labeled by spin (*s* = ±1) and valley pseudospin (*l* = ±1). The model we use to describe carbon nanotube quantum dots (CNTQDs) is an extended two-orbital Anderson model:

[1]
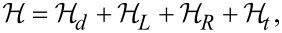


where the dot Hamilonian reads:

[2]
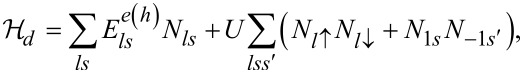


with site dot energies:

[3]
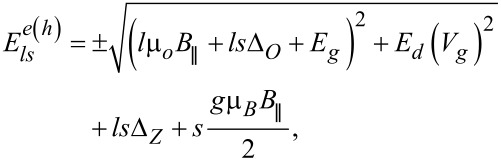


depending on the magnetic field *B*_∥_ and the gate voltage *V_g_*. The upper and lower signs, ±, refer to conduction or valence states, μ*_o_* is orbital magnetic moment μ*_o_* = *ev*_F_*D*/4, where *v*_F_ is the Fermi velocity (*v*_F_ ≅ 0.8*c*), *D* is the nanotube diameter,


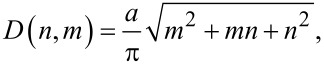


and *a* is the distance between carbon atoms in the A lattice and the B lattice of graphene (*a* ≅ 0.254 nm). *E_g_* is the bandgap energy,


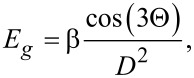


where Θ is the chiral angle, 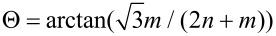. According to tight-binding calculations the value of β corresponding to the equilibrium energy gap is 37 meV·nm^2^ [9,63]. Δ*_O_* and Δ*_Z_* stand for orbital and Zeeman parameters of spin–orbit coupling in the form:

[4]
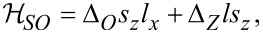


where *s_z_* is the spin component along the nanotube axis and *l_x_* is the Pauli matrix in the A–B graphene sublattice space. Δ*_Z_* = −δcos(3Θ)/*D* and Δ*_O_* = δ/*D* [63]. Various theoretical and experimental estimates differ not only in the reported values of the parameter δ but also often in the predictions of its sign. It ranges from one tenth to a few meV·nm [42]. For wide-bandgap nanotubes, *E_g_* ≫ Δ*_Z_*, Δ*_o_*, the field dependence of single-particle energies (Equation 3) becomes linear,


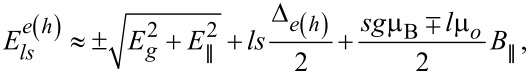


for small-bandgap nanotubes it is parabolic. For wide-bandgap nanotubes, SO splitting can be described by one effective parameter:


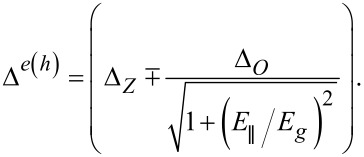



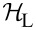
 and 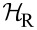 describe electrons in the left and in the right electrode, respectively:

[5]
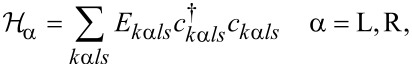


and the last term in Equation 1 represents tunneling:

[6]
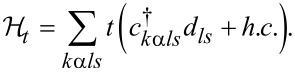


We parameterize the coupling strength to the leads by Γ = 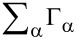 = 
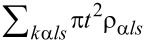
. In the following, the wide-conduction-band approximation with a rectangular density of states is used, ρ_α_*_ls_*(*E*) = 1/2*W*, where *W* is the half-bandwidth.

To analyze correlation effects we use the slave-boson mean-field (SBMFA) approach of Kotliar and Ruckenstein (K-R) [26,64–65]. In this picture, different auxiliary bosons are introduced to project onto different orbital and spin states. Apart from empty-state bosons, *e*, singly occupied *p_ls_*, doubly occupied *d*, triply occupied *t_ls_*, and fully occupied *f* bosons are used. The *p* operators are labeled with indices specifying the corresponding single-particle states, *t* bosons are characterized by hole indexes, and six *d* operators project onto doubly occupied states *d_l=_*_±1_: |20⟩, |02⟩ and *d_ss_*_′_: |↑↑⟩, |↓↓⟩, |↑↓⟩ and |↓↑⟩. To eliminate unphysical states, the completeness relations for the slave-boson operators,


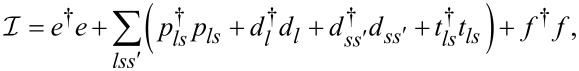


and the conditions for the correspondence,


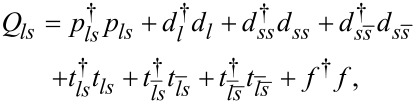


between fermions and bosons have to be imposed. These constraints can be enforced by introducing Lagrange multipliers λ and λ*_ls_*. In the K-R approach, the Hamiltonian in Equation 1 describing interacting fermions is replaced by an effective Hamiltonian of noninteracting bosons and pseudofermions. It takes the form:

[7]
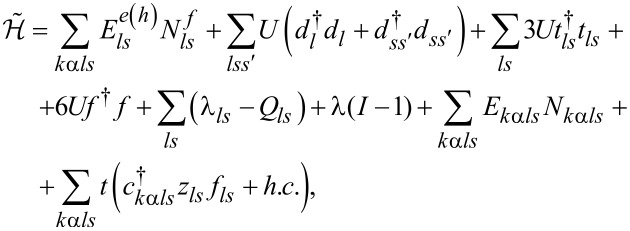


where 
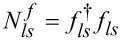
 are the pseudofermion occupation operators and *f_ls_* is defined by *f_ls_* = *d_ls_z_ls_* with the boson operator


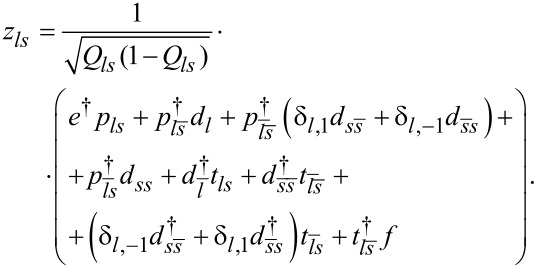


The mean-field solutions are found from the minimum of the free energy with respect to the mean values of slave-boson operators and Lagrange multipliers. The SBMFA method is correct in the unitary Kondo regime and it leads to a local Fermi-liquid behavior at zero temperature. It gives reliable results of the linear conductance also for systems with weakly broken symmetry. The results obtained are in good agreement with experiments and with renormalization group calculations [27–28]. The SBMFA method best describes systems close to the Kondo fixed point, that is, for the case of deep fully degenerate atomic levels at low temperatures. This approximation correctly describes spin or pseudospin fluctuations and reproduces local Fermi-liquid behavior at zero temperature. It breaks down at higher temperatures. The reason why mean-field approximation (MFA) results get worse with increasing temperature is the neglect of thermal fluctuations, which violate the constraint conditions, as they are only imposed on average in SBMFA. The disadvantage of MFA is that it breaks the gauge invariance symmetry, which is associated with charge conservation. Consequently, at higher temperatures, a spurious transition to the local-moment regime is found rather than a continuous crossover. When analyzing higher temperatures, one can avoid the high-temperature drawbacks of SBMFA by including fluctuations of boson fields, for example, by performing a 1/*N* expansion around the mean-field solutions. This can be done, for instance, following the schemes drawn in [66–67] or using slave-boson techniques that are not of mean-field nature [68–70]. Both approaches describe also charge fluctuations in addition to spin fluctuations. Alternatively, one can use another complementary many-body technique that correctly predicts in the high-temperature range. Technically simple is the equation of motion (EOM) method with Lacroix truncation of the chain of Green functions at the second order in the hybridization term [71]. In this approach, which is applied by us, one approximates the Green functions involving two conduction-electron operators by:

[8]
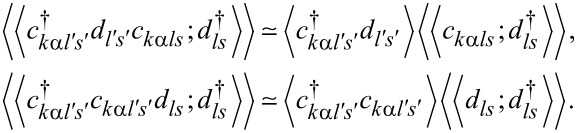


The correlations 
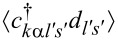
 and 
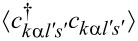
 are most important for the Kondo effect. Lacroix EOM accounts not only for spin or pseudospin fluctuations, but also for charge fluctuations. One of the most appealing features of this approximation is that it can work in the whole parameter space except for the close vicinity of the Kondo fixed point. The described method breaks down at low temperatures. Deficiencies of the Lacroix approximation are that this method does not recover Fermi-liquid relationships and that the Kondo effect is absent at the particle–hole symmetry point. Simple higher-order truncations remove the latter drawback [72]. There are also attempts to recover Fermi-liquid behavior at low energy in the EOM formalism [73] but, to our knowledge, without full success.

The two physical quantities that are the object of our interest are linear conductance 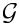 and thermoelectric power (TEP) 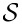. Both quantities can be determined from the transmissions, which, in turn, can be calculated from the knowledge of Green’s functions obtained in SBMFA or EOM:


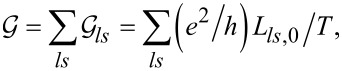



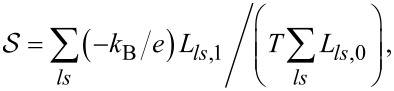


where


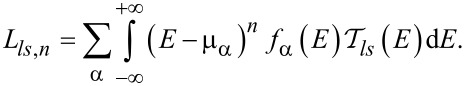


*f*_α_(*E*) is the Fermi distribution function of electrodes and μ_α_ = ±*V_sd_*/2. 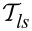 is the spin–orbital transmission. Important quantities for spintronics and valleytronics are spin (SPC) or orbital (OPC) polarizations of conductance directly expressed through partial conductances,


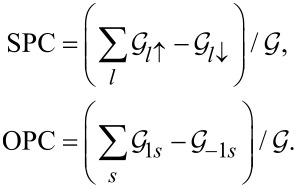


Spin and orbital magnetic moments are defined as


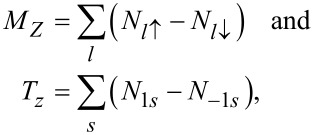


where *N_ls_* denotes electron occupations.

## Results and Discussion

**Figure 1:**
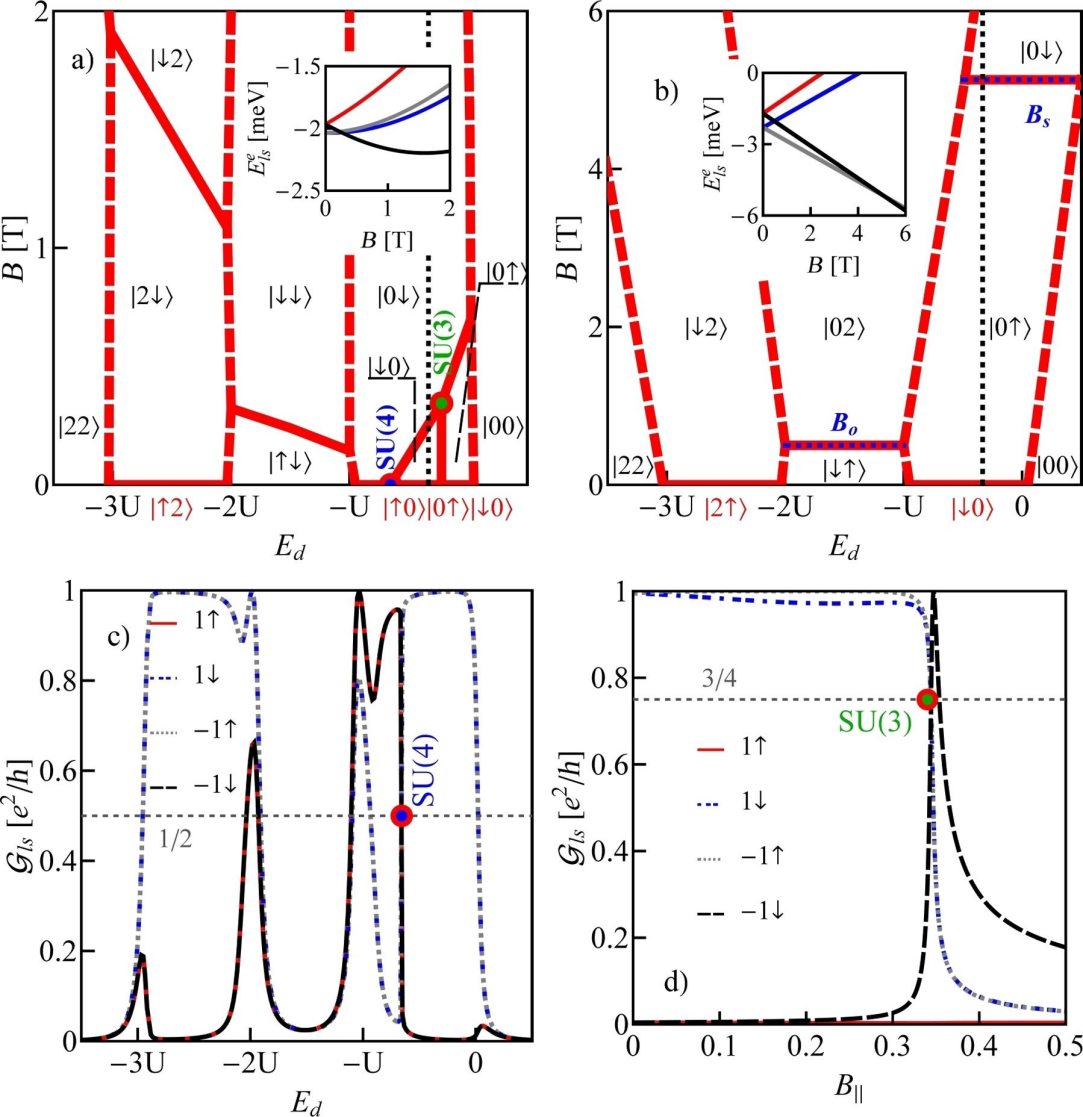
(a, b) Ground-state maps of isolated quantum dots (*U* = 6 meV, δ = 1 meV·nm): (a) dot formed in a small-bandgap nanotube, C(24,21) *E_g_* = 0.45 meV (this bandgap energy corresponds to β = 37 meV·nm^2^), (b) dot in a semiconducting tube, C(24,22) (*E_g_* = 125 meV). The dashed red lines are the borders between Coulomb blockade valleys and the solid lines are double-degeneracy lines. Blue and green points denote threefold- and fourfold-degeneracy points, respectively. The black brackets |*ls*⟩ represent the ground states for *B >* 0 and brackets in red are the ground states for *B <* 0. Insets show the dependencies on the magnetic field of energies of the single-particle states |↑0⟩, |↓0⟩, |0↑⟩ and |0↓⟩ (red, blue, gray and black lines, respectively) plotted for the cross sections denoted by the dotted black vertical lines. (c) Partial-conductance values of the dot CNTQD(24,21) strongly coupled to the leads for *B_||_* = 0. (d) Conductance values for fixed values of the gate voltage (cross section through the SU(3) point specified by the dotted black line, Γ = 0.03 meV).

All numerical results presented below concern quantum dots formed in nearly metallic nanotubes with perturbation gaps. We compare in Figure 1 the ground-states diagram of an isolated quantum dot formed in a small-bandgap nanotube, C(24,21) (*n* = 24, *m* = 21, bandgap energy *E_g_* = 0.46 meV, Figure 1a) with the diagram of a dot in a wide-bandgap nanotube, C(24,22) (bandgap *E_g_* = 125 meV, Figure 1b). The insets present field dependencies of single-electron states, which, according to Equation 3, are linear for wide-bandgap nanotubes and parabolic for nanotubes with narrow bandgaps. For vanishing magnetic fields, the ground states in odd Coulomb valleys are degenerate (Kramers degeneracy). For the assumed SO parameters (Δ*_Z_* = −0.02 meV, Δ*_O_* = −0.32 meV) (β = 37 meV·nm^2^) the ground-state doublet is {|0↑⟩,|↓0⟩} in the 1e valley and {|↓2⟩,|2↑⟩} in the 3e valley. In even valleys, the ground state is singlet |↓↑⟩. Magnetic fields break the time-inversion symmetry, which results in a splitting of Kramers doublets in odd valleys. Depending on the signs of SO coupling the recovery of degeneracy resulting from a competition of Zeeman effect and SO interaction can result in 1e or 3e valleys [9]. For the analyzed example of a wide-bandgap nanotube, a crossing of energy levels occurs in the 1e valley. For a field of *B_s_* = |Δ*_e_*|/gμ_B_ ≈ 5.17 T, the energy of the state |0↑⟩ is crossed by the energy line of one of the states from a higher Kramers doublet |0↓⟩ (inset in Figure 1b) and degeneracy is recovered. The characteristic field is determined by SO splitting alone and, therefore, the degeneracy line is parallel to the gate-voltage axis. Recovery of degeneracy is also observed in the 2e valley, that is, the two states |↓↑⟩ and |02⟩ degenerate in a magnetic field of *B_o_* = |Δ*_e_*|/μ*_o_* ≈ 0.48 T. In small-bandgap nanotubes, the field dependencies of degeneracy lines are determined not only by spin–orbit parameters and orbital and spin magnetic moments, but also by the gap and the gate voltage. This is reflected in the nonlinear gate dependencies of degeneracy lines and the possibility of degeneracy of more than two states. The fact that the boundaries between areas of different ground states are not parallel to the gate axis opens the path for electric control of transitions between different ground states. Consequently, it also enables switching of physical quantities such as magnetic or orbital moments of the dots (examples of the maps of magnetic and orbital moments are given below in Figure 3c,d). The occurrence of points of different degeneracy and the ability to move between them by change of the gate voltage may have significance for quantum computing by providing a method for electrically switching, in the same nanoscopic system, between spin, valley, or spin–valley qubits, as well as between qubits and qutrites (threefold degeneracy) or qudits (fourfold degeneracy) of various types. For the analyzed nanotube C(24,21), threefold and fourfold degenerate points appear in the 1e valley. The SU(3) point is observed for finite field in the single-occupancy region and fourfold degeneracy occurs in the same valley for zero field.

Till now, we described the properties of isolated CNTQDs. Now, we want to concentrate on their transport properties in the strong-correlation regime. Figure 1c,d presents partial-conductance values of a dot formed in a nanotube C(24,21), CNTQD(24,21), for the case that the dot is strongly coupled to the leads and Kondo effects occur at degeneracy points or lines. Figure 1c presents gate-dependencies of partial conductances for zero magnetic field, where the SU(4) spin–orbital Kondo point separates intervals of occurrence of SU(2) Kondo effects related to two different Kramers doublets. For lower gate voltages, the {|↑0⟩,|0↓⟩} states are active in cotunneling processes and for higher voltages the states {|↓0⟩,|0↑⟩} are active. Consequently, transport channels labeled by these quantum numbers are active with conductance values per channel close to the unitary limit *e*^2^/*h* for SU(2) lines and (1/2)(*e*^2^/*h*) per channel at the SU(4) point. Figure 1d illustrates partial conductances for the field-induced SU(3) Kondo effect. The curve is drawn for a fixed value of the gate voltage. The partial conductance of each of the three channels takes a value of (3/4)(*e*^2^/*h*).

**Figure 2:**
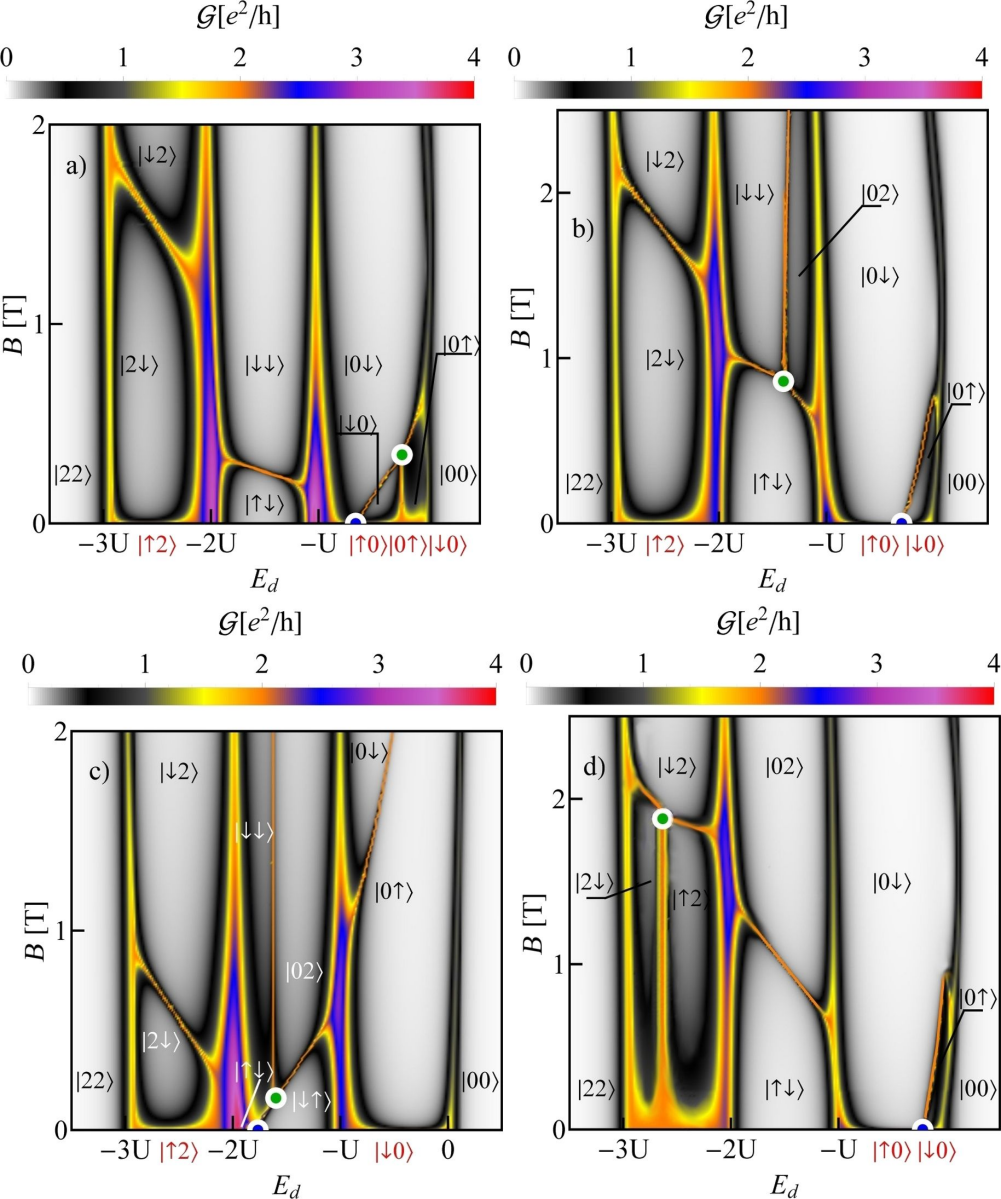
Maps of the total conductance values with plotted ground-state diagrams of (a) CNTQD(24,21), (b) CNTQD(39,24), (c) CNTQD(15,12), and d) CNTQD(48,18) (*U* = 6 meV, Γ = 0.03 meV, δ = 1 meV·nm, β = 37 meV·nm^2^).

In the example discussed above, the high-symmetry points are located in the region of single occupation, but in different nanotubes they can be located in different occupation areas. For brevity of presentation we show in Figure 2 only four examples of conductance maps of CNTQDs differing in the mutual positions of high-symmetry points. SU(4) points appear from the intersection of four SU(2) lines and this occurs in zero magnetic field. This is a general condition resulting from the time-inversion symmetry. The appearance of a SU(4) Kondo state in nanotubes with finite SO interaction is a surprising result. It occurs because of the gate-induced reconstruction of the dot states, which, for a certain gate voltage, compensates the changes induced by SO coupling. The SU(3) Kondo effect is field-induced and a threefold-degeneracy point occurs at the intersection of three lines of double degeneracy. To indicate which quantities fluctuate in the presented Kondo effects, we have also marked in Figure 2 the corresponding ground states appearing in different areas of the maps. The degeneracy lines corresponding to the same occupation are the borders between different ground states in a given Coulomb valley and the effective fluctuations of these degenerated states, induced by cotunneling processes, lead to the formation of Kondo resonances.

The first map (Figure 2a) refers to the CNTQD(24,21) discussed above. However, now the conductance is shown for a wide range of the magnetic field and over the entire range of occupation of the first shell. Different gate-dependent SU(2) conduction lines, reaching values close to 2*e*^2^/*h*, are seen. In the 1e region there are a spin Kondo effect with the fluctuating states |0↓⟩ and |0↑⟩ exhibiting orbital polarization, a valley Kondo effect with the |0↓⟩ and |↓0⟩ states exhibiting spin polarization, and a spin–valley Kondo effect with fluctuations of |↓0⟩ and |0↑⟩, in which both spin and orbital moments are quenched. The SU(3) Kondo effect is caused by cotunneling-induced fluctuations of |0↓⟩, |0↑⟩, and |↓0⟩ states and the resulting resonance is spin- and orbital-polarized. SU(4) Kondo screening results from effective fluctuations of all four spin–orbital states |*ls*⟩.

**Figure 3:**
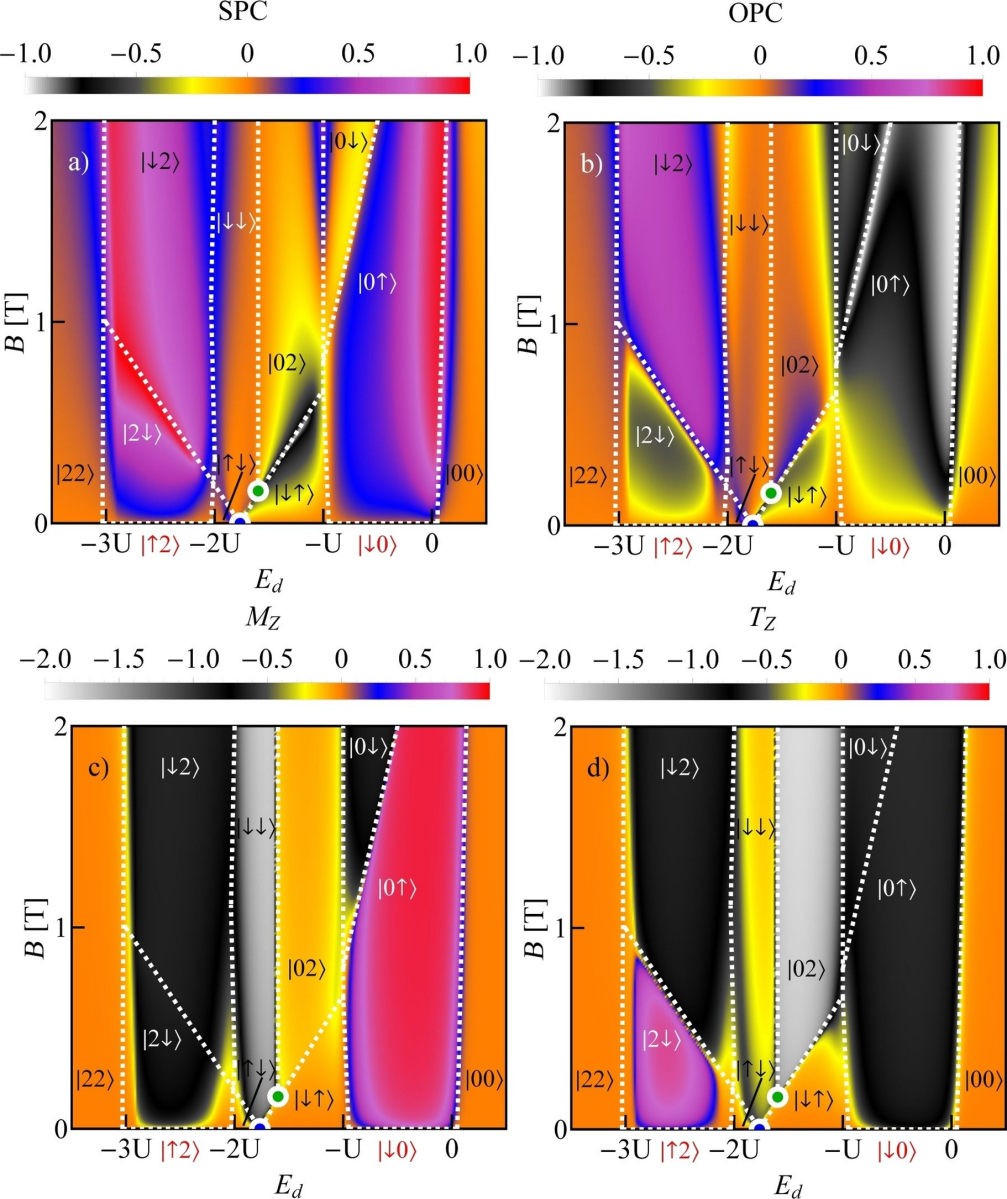
(a, b) Spin polarization (SPC) and orbital polarization (OPC) maps of CNTQD(15,12). (c, d) Spin (*M_Z_*) and orbital pseudospin (*T_Z_*) magnetization diagrams of CNTQD(15,12). Dotted white lines represent degeneracy lines.

In the 2e valley, we observe a two-electron spin-polarized spin Kondo effect with |↑↓⟩ and |↓↓⟩ dot states engaged. In the 3e valley, there is a spin–orbit effect with fluctuating |↓2⟩ and |2↓⟩ states and screened spin and orbital moments. On the next map representing the conductance of a quantum dot formed in C(39,24), the tube SU(4) point lies in the 1e valley and the SU(3) point is in the region of double occupancy (Figure 2b). In Figure 2c, which presents the conductance of CNTQD(15,12), both SU(4) and SU(3) points are located in the 2e valley. The last map (Figure 2d), presenting the conductance of CNTQD(48,18), has the SU(4) point in the 1e valley and the SU(3) point in the 3e valley. As already mentioned, the examples presented above do not exhaust all possible locations of high-symmetry points. Figure 3a presents an example of spin polarization of conductance corresponding to the conduction map in Figure 2c. An interesting feature regarding spintronics is the occurrence of Kondo lines with high positive and negative conductance polarizations, between which one can switch through changing the gate voltage (spin filter).

**Figure 4:**
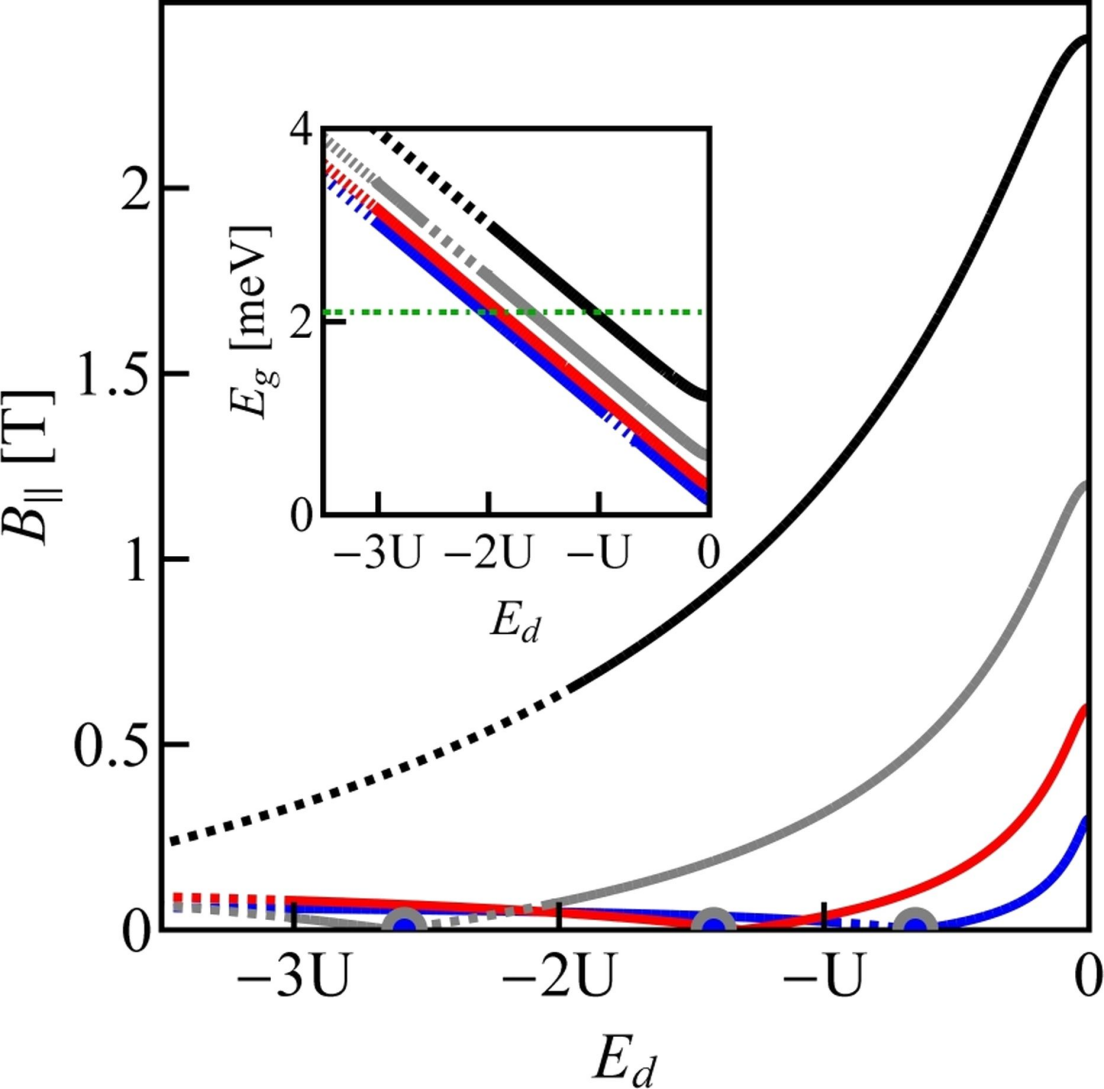
SU(3) Kondo lines of CNTQD(15,12) as functions of magnetic field and on-site energy plotted for several SO parameters. The inset shows SU(3) lines as function of on-site energy and bandgap. Solid lines correspond to SU(3) Kondo states and dotted parts of the lines indicate the regions where Kondo correlations are destroyed. The green horizontal dotted line in the inset shows the equilibrium value of the bandgap for the unstrained nanotube. Blue points represent the SU(4) high-symmetry Kondo solution (*B*_∥_ = 0). Blue, red, gray and black lines are for δ = 1/4, 1/2, 1, and 2 meV·nm (*U* = 6 meV).

For any narrow-bandgap nanotube, one can move the SU(3) point between different occupation areas by changing the magnetic field and the value of the bandgap; the latter change can be induced by strain. The examples of SU(3) lines drawn for CNTQD(15,12) for several assumed SO parameters are presented in Figure 4. The required magnetic field for the occurrence of threefold degeneracy for a given gate voltage can be read from the main picture, while the bandgap can be read from the inset. The solid lines present SU(3) Kondo solutions and the dotted parts of the lines correspond to the situation where Kondo correlations are destroyed. The fourfold-degeneracy points are also marked and they appear when the SU(3) line touches the *B* = 0 line. Knowing the set of parameters specifying the given nanotube (bandgap, Zeeman-like and orbital-like spin–orbit coupling parameters, and spin and orbital magnetic moments) one can determine the dot energy and magnetic field for which the state SU(3) will occur. The corresponding expressions are given below in Equation 9 and Equation 10; here μ*_s_* = *g*μ_B_ is the spin magnetic moment.

[9] $$E_{d}^{\text{SU(3)}}=\frac{\sqrt{\frac{\Delta_{Z}^{2}\left(-E_{g}^{2}+\Delta_{Z}^{2}\right)\left(-E_{g}\mu_{o}+\Delta_{Z}\mu_{s}+\Delta_{O}\left(\mu_{o}-\mu_{s}\right)\right)\left(-\mu_{o}^{2}+\mu_{s}^{2}\right)\left(-E_{g}\mu_{o}+\Delta_{Z}\mu_{s}+\Delta_{O}\left(\mu_{o}+\mu_{s}\right)\right)}{\mu_{s}^{2}{\left(E_{g}\mu_{o}-\Delta_{Z}\mu_{s}\right)}^{2}}}}{\Delta_{Z}},$$

[10] $$B_{\|}^{\text{SU(3)}}=\left|\frac{E_{g}\mu_{o}\Delta_{Z}-\Delta_{O}\Delta_{Z}\mu_{o}+E_{g}\mu_{s}\Delta_{O}-\Delta_{Z}^{2}\mu_{s}}{E_{g}\mu_{o}\mu_{s}-\Delta_{Z}\mu_{s}^{2}}\right|$$

SU(4) state occurs for *B* = 0 and

[11]
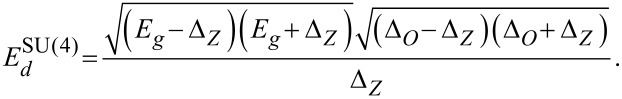


**Figure 5:**
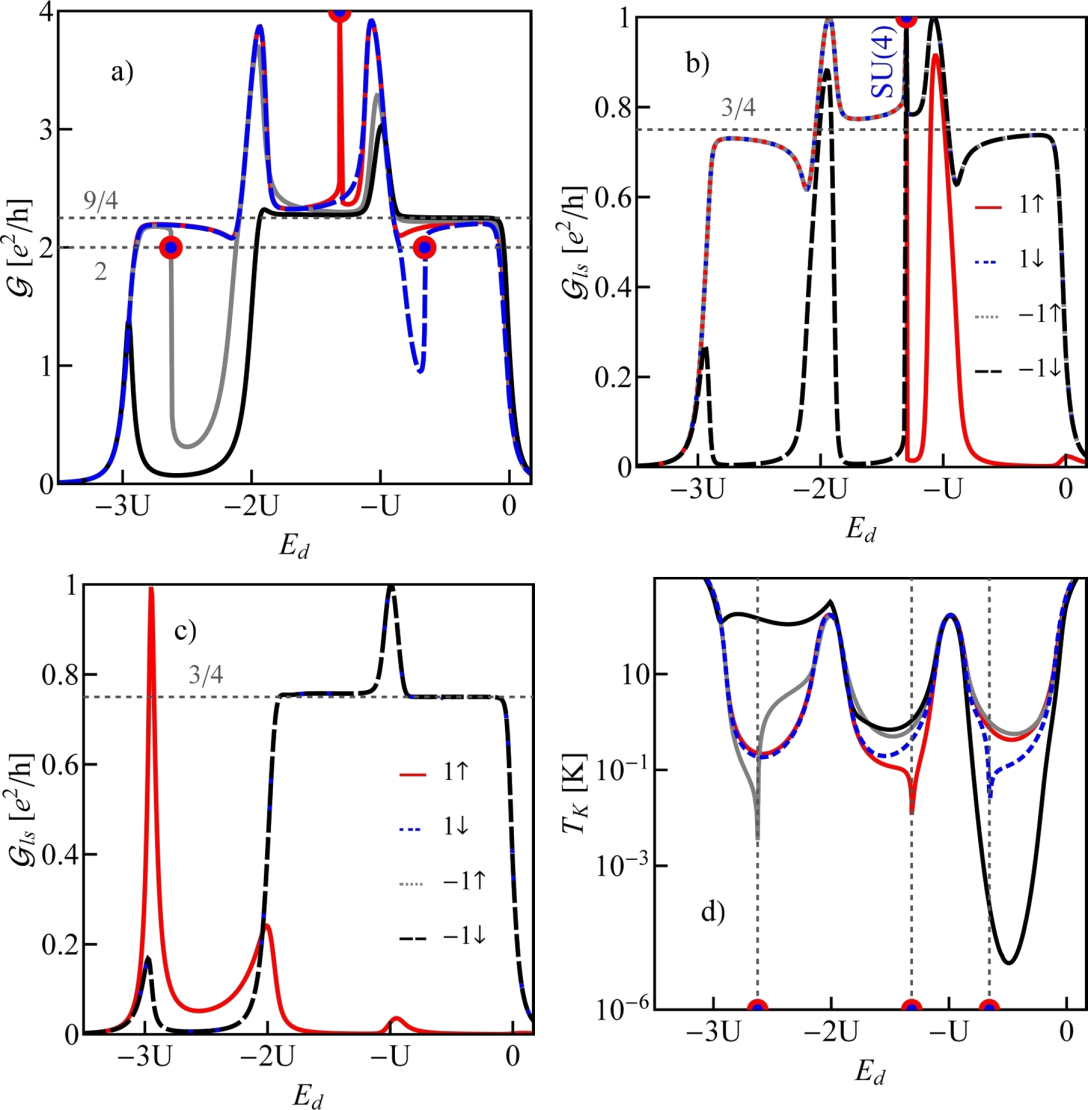
(a, d) Total conductances (a) and Kondo temperature (d) of CNTQD(15,12) plotted as a function of the on-site energy. Blue, red, gray and black lines represent values of δ = 1/4, 1/2, 1, and 2 meV·nm, respectively. b) Partial conductances for δ = 1/2 meV·nm. c) Partial conductances for δ = 1 meV·nm (*U* = 6 meV, Γ = 0.03 meV).

Equations 9–11 are satisfied when *E_g_* *>* Δ*_Z_* and Δ*_O_* *>* Δ*_Z_*. The gate dependence of conductance values corresponding to the states in Figure 4 are shown in Figure 5. Horizontal dashed lines indicate the characteristic limit of the SU(3) Kondo conductance, 
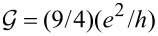
, and the unitary value of the SU(4) Kondo effect in odd valleys 
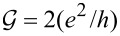
. The centers of Kondo resonances are shifted from *E*_F_ in odd valleys, that is, they are shifted towards lower gate voltages in the 1e valley and towards higher voltages in the 3e valley. In the 2e valley, the conductance of the SU(4) Kondo point reaches a value of 
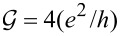
 due to the presence of six degenerate states. A Kondo state is formed due to cotunneling-induced fluctuations between all these states. The SU(4) Kondo resonance, in this case, is centered at *E*_F_. For gate-voltage intervals in which Kondo correlations are destroyed a drop of conductance is observed. We also present examples of partial conductances for two values of SO coupling δ = 0.5 meV·nm, for which the SU(4) point is located in the 2e valley and δ = 1 meV with the SU(4) point in the 3e region. In the latter case we observe partial SU(3) conductances in the 1e and 2e valleys reaching almost a value of (3/4)(*e*^2^/*h*), identical for the three spin channels. For δ = 0.5 meV·nm, the SU(4) point divides the Kondo SU(3) line in the 2e region into two parts associated with two different resonances of the same symmetry. One SU(3) resonance associated with fluctuations of the states {|↓↑⟩, |↓↓⟩, |02⟩} and the other with fluctuations of {|↑↓⟩, |↓↓⟩, |02⟩}. The corresponding partial conductances, equally contributing to the total conductance, are 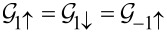 = (3/4)(*e*^2^/h) for the left-hand side resonance and 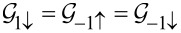 = (3/4)(*e*^2^/*h*) for the right-hand side resonance (Figure 5b). The fact that SU(3) Kondo states occur on both sides of the SU(4) point is also seen by the red solid line in Figure 5a.

**Figure 6:**
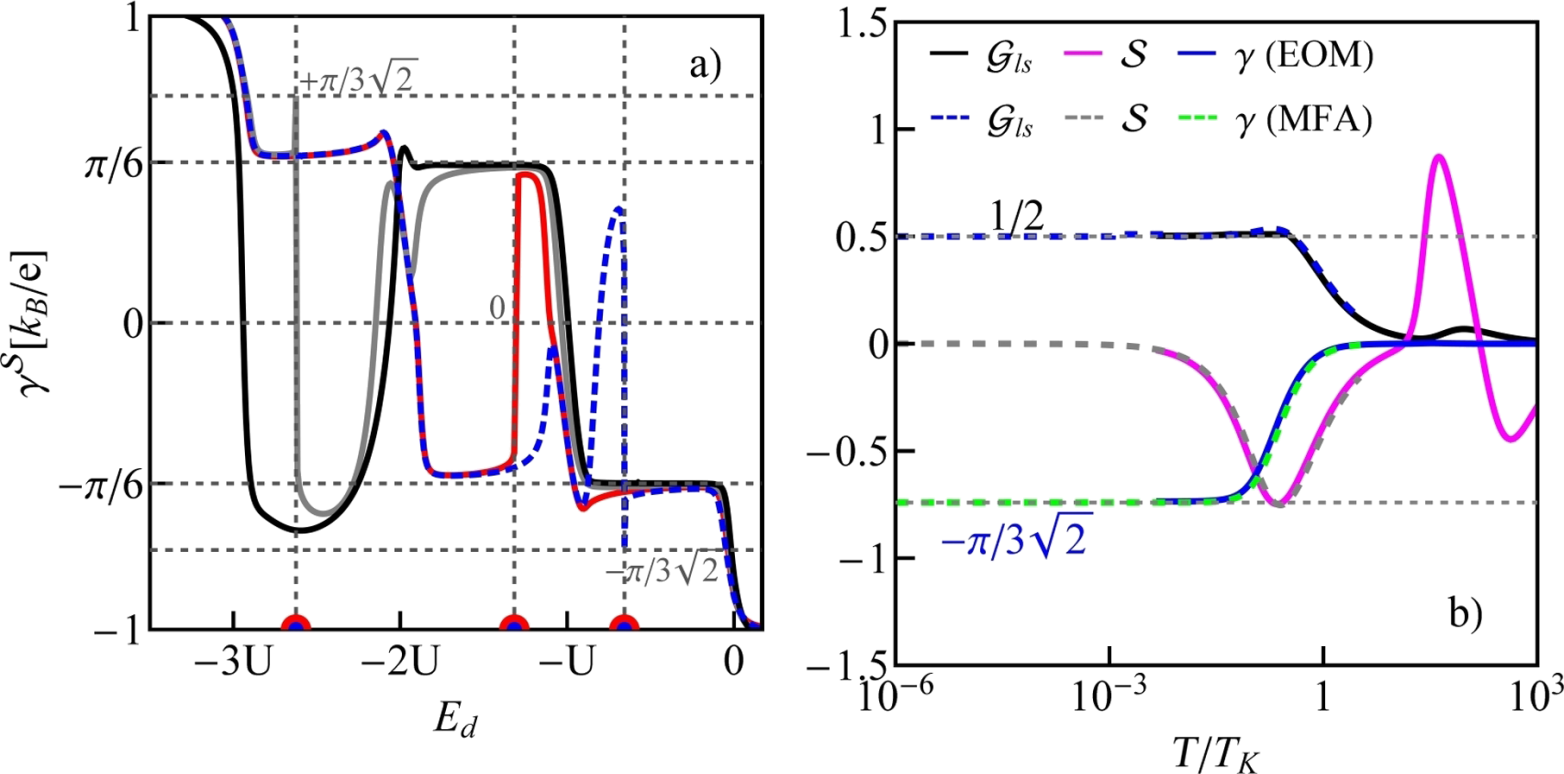
Thermoelectric quantities and conductance of CNTQD(15,12). (a) Linear TEP coefficient 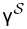 as a function of *E_d_*. Blue, red, gray and black lines are for δ = 1/4, 1/2, 1, and 2 meV·nm, respectively. (b) Partial conductance 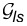, TEP (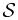), and 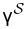 as functions of the normalized temperature *T*/*T*_K_ for δ = 1/4 meV·nm. The dotted and solid lines present results of the SBMFA and the EOM method, respectively (*U* = 6 meV, Γ = 0.03 meV, β = 37 meV·nm^2^).

**Figure 7:**
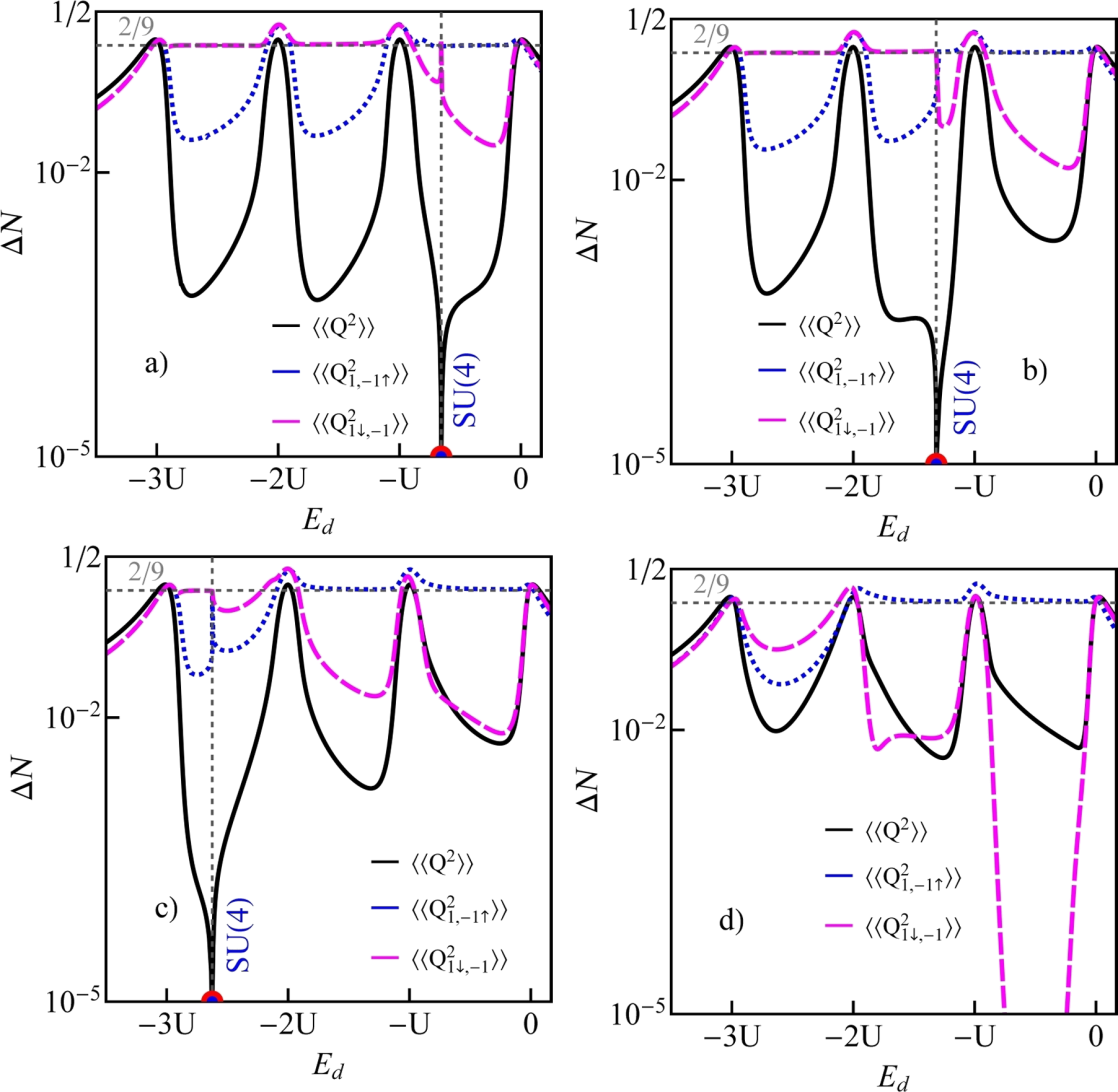
Charge and spin–orbital fluctuations of CNTQD(15,12) plotted for (a) δ = 1/4 meV·nm, (b) δ = 1/2 meV·nm, (c) δ = 1 meV·nm, and (d) δ = 2 meV·nm (*U* = 6 meV, Γ = 0.03 meV, β = 37 meV·nm^2^).

The quantity that clearly reflects the symmetries of the many-body resonances and their electron or hole character within the shell is a linear thermoelectric coefficient of the thermopower defined as [74]


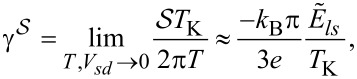


where the Kondo temperature *T*_K_ is given by the center and the width of the Kondo resonance,


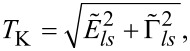


with


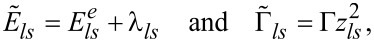


where *ls* labels the dot states active in the Kondo processes [64]. Plots of 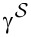 are presented in Figure 6a. The dashed gray horizontal lines correspond to the characteristic limits for a given symmetry, ±π/6(*k*_B_/*e*) for the SU(3) Kondo effect and 
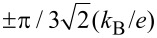
 for SU(4) in odd valleys. In the 1e valley, regardless of symmetry, 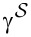 is always negative (electron nature), and in the 3e valley it is positive (hole character). In the 2e valley 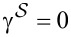 for the SU(4) Kondo state. For SU(3) symmetry, a change of the sign of 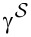 is observed, which reflects the transition between the previously mentioned two SU(3) Kondo states. Switching between different Kondo SU(3) states is also visible in the spin–orbital fluctuations associated with the given resonances. At this point it should be mentioned that one should look at the high-temperature results in Figure 6b with some caution, in particular, at those that concern the thermopower. Inelastic processes, for example, these resulting from electron–phonon interaction are neglected in our calculations, which is justified at extremely low temperatures. At higher temperatures, however, they may play a role [75–76]. Some justification for the neglect of electron–phonon coupling is that, as shown in [77–80], the electron–phonon mean free path in nanoscale carbon tubes and ribbons is tens of micrometers even at room temperature. It has been also shown that the phonon contribution to the thermopower of quantum dots is greatly suppressed [81]. The Landauer-type formulas we use strictly apply only to elastic transport. The electron part of the thermopower is very sensitive to the details of the spectral function, which is strongly affected by correlations. We believe that even in the crude Landauer approximation, our qualitative results on thermopower still provide important information about resonances that play a role at different temperatures. Figure 7 presents charge fluctuations and spin–orbital fluctuations corresponding to two types of SU(3) Kondo effects occuring in the system. The corresponding second cumulants are defined as follows:

[12] $$\begin{array}{c} \langle \langle Q^{2}\rangle \rangle =\langle {\left(N-\langle N\rangle \right)}^{2}\rangle =\langle N^{2}\rangle -{\langle N\rangle }^{2},\\ \langle \langle Q_{1,-1↑}^{2}\rangle \rangle =\langle \sum_{s}{\left(N_{1s}+N_{-1↑}\right)}^{2}\rangle -{\langle \sum_{s}\left(N_{1s}+N_{-1↑}\right)\rangle }^{2},\\ \langle \langle Q_{1↓,-1}^{2}\rangle \rangle =\langle \sum_{s}{\left(N_{1↓}+N_{-1s}\right)}^{2}\rangle -{\langle \sum_{s}\left(N_{1↓}+N_{-1s}\right)\rangle }^{2}. \end{array}$$

The above fluctuations can be easily expressed by slave-boson operators:

[13] $$\begin{array}{c} \langle \langle Q^{2}\rangle \rangle =\sum_{ls}p_{ls}^{2}+4\sum_{lss^{\prime }}\left(d_{l}^{2}+d_{ss^{\prime }}^{2}\right)+9\sum_{ls}t_{ls}^{2}+16f^{2}-\\ -{\left[\sum_{ls}p_{ls}^{2}+4\sum_{lss^{\prime }}\left(d_{l}^{2}+d_{ss^{\prime }}^{2}\right)+9\sum_{ls}t_{ls}^{2}+16f^{2}\right]}^{2}, \end{array}$$

[14]
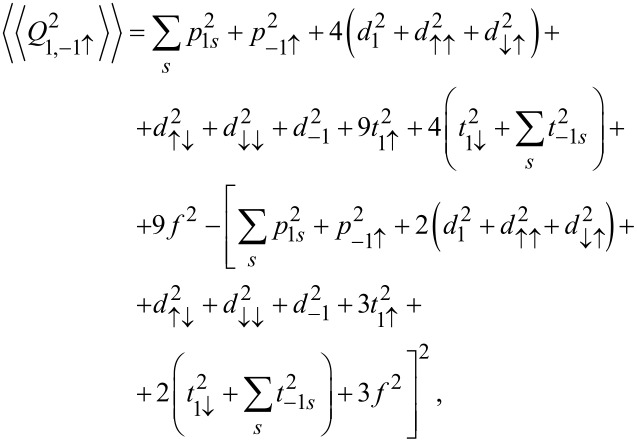


[15]
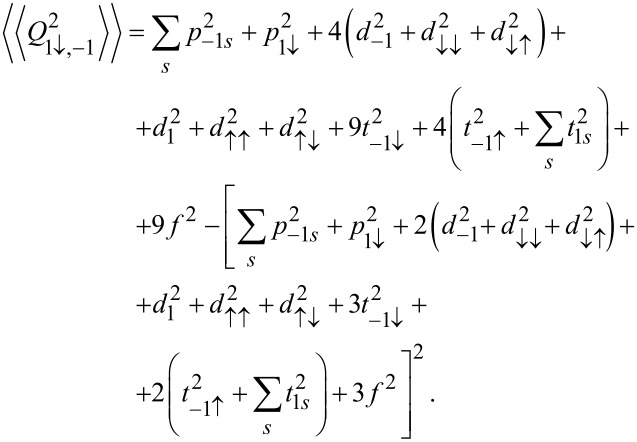


Charge fluctuations and the spin–orbit fluctuations that relate only to the dot states active in Kondo processes have small values and are characterized by clear minima in the regions in which the SU(3) resonance occurs. An interesting observation is that the gate dependence of the Kondo temperature qualitatively resembles the dependencies of these fluctuations (Figure 7, Figure 5d). Other spin–orbital fluctuations, not related solely to active states in Kondo processes, weakly depend on the gate voltage and the value they take depends on the symmetry.

Figure 6b shows the temperature dependencies of conduction, thermoelectric power, and the coefficient 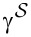 given for the SU(4) point in the 1e valley of CNTQD(15,12). The values were calculated using SBMFA and, additionally, the EOM method with Lacroix approximation [71]. The latter approach is introduced to account for the high-temperature behavior. At low temperatures, both methods reproduce limits characteristic for the SU(4) resonance. At *T* ≪ *T*_K_, the TEP approaches a local minimum. At higher temperatures, it increases and changes its sign. This signals the disappearance of Kondo correlations. At even higher temperatures, another minimum of the TEP is observed, which is due to Coulomb resonance.

**Figure 8:**
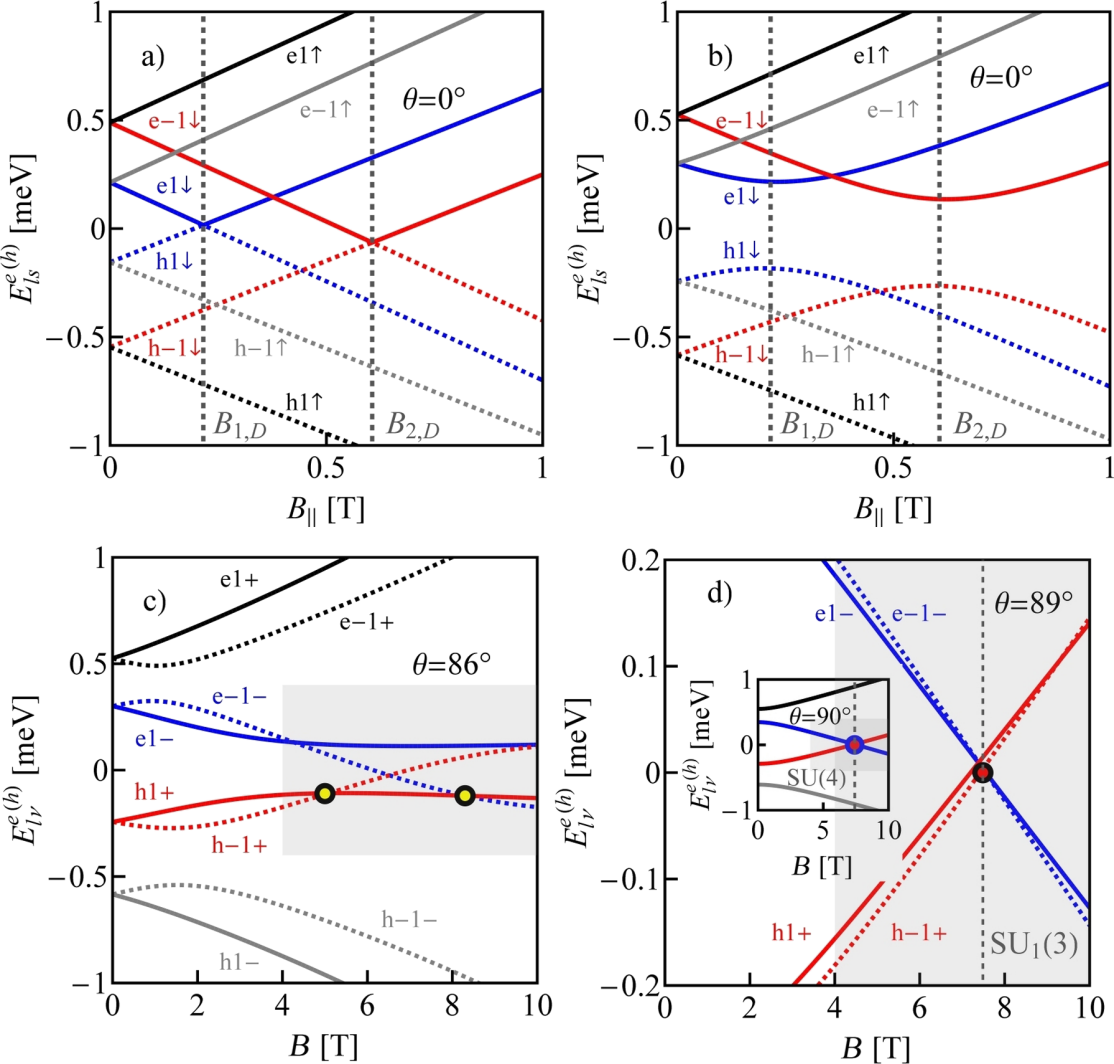
Electron and hole states of (a) a carbon nanotube C(33,30) and of (b–d) a quantum dot CNTQD(33,30), in (b) a parallel magnetic field (θ = 0°) and in slanting magnetic fields of (c) θ = 86°, and (d) θ = 89° (δ = 3/2 meV·nm, β = 37 meV·nm^2^, *E_d_* = −0.2 meV).

In nearly metallic carbon nanotubes, magnetic fields of several teslas close the bandgap. An example of the field dependencies of the lowest electron and the highest hole states of the nanotube C(33,30) is presented in Figure 8a. The fields at which the bandgap closes are called Dirac fields. In CNTQDs, parallel magnetic fields do not close the bandgap due to a finite confinement energy. Figure 8b shows the field dependencies of four states from the lowest electron shell and four states from the highest hole shell of the quantum dot CNTQD(33,30). No crossing of the electron line with the hole line is observed for any value of the parallel magnetic field (θ = 0°). It is noteworthy that the minima or maxima appearing in the field dependencies of electron or hole states of quantum dots formed in a given nearly metallic nanotube occur for fields equal to the Dirac fields of the corresponding infinite nanotube. Crossing of electron and hole energy lines is observed in slanting fields (Figure 8c,d). For non-parallel fields, the spin states |↑⟩ and ⟨↓| are mixed by the perturbation


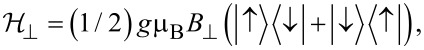


where *B*_⟂_ = *B*·sin(θ). We will denote the new spin states by |+⟩ and |−⟩. The states are also labeled by the orbital index *l* and we additionally introduce in the designation of states the letters “e” and “h” to distinguish between electron and hole states. In this notation the single-particle dot states of interest are |e1−⟩, |e−1−⟩, |h1+⟩, and |h−1+⟩.

Although we discuss in the following many-body resonances only for the specific example of CNTQD(33,30), the analysis and conclusions presented below apply to all dots formed in nearly metallic nanotubes. Figure 8c,d shows the field dependencies of electron and hole energies for θ = 86°, θ = 89°, and, in the inset of Figure 8d, additionally for θ = 90°. For θ = 86°, two ground-state double-degeneracy points are observed. There is a hole–hole degeneracy point at lower fields (|h1+⟩, |h−1+⟩) and an electron–hole degeneracy at higher fields (|h1+⟩, |e−1−⟩). Figure 8d shows the case of threefold degeneracy |e1−⟩, |e−1−⟩, |h−1+⟩ and the inset of Figure 8d illustrates a fourfold electron–hole degeneracy occurring in transverse fields (|h1+⟩, |h−1+⟩, |e1−⟩, |e−1−⟩).

**Figure 9:**
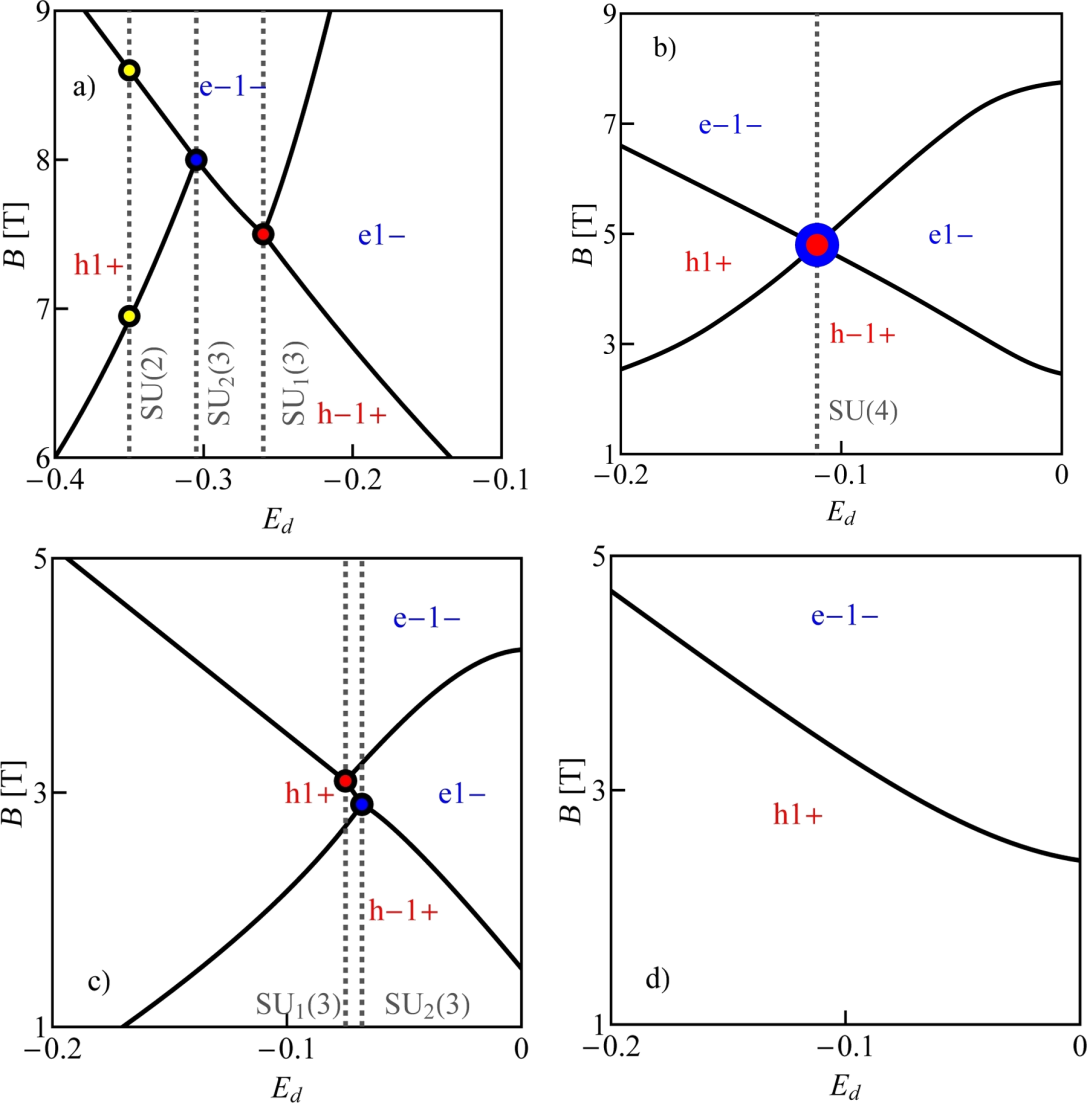
Electron–hole ground-state diagrams of CNTQD(33,30) in a slanting magnetic field (θ = 89°) for different SO parameters: (a) δ = 3/2 meV·nm, (b) δ = 0.9 meV·nm, (c) δ = 0.8 meV·nm, and (d) δ = 0.4 meV·nm (*U* = 6 meV, β = 37 meV·nm^2^).

**Figure 10:**
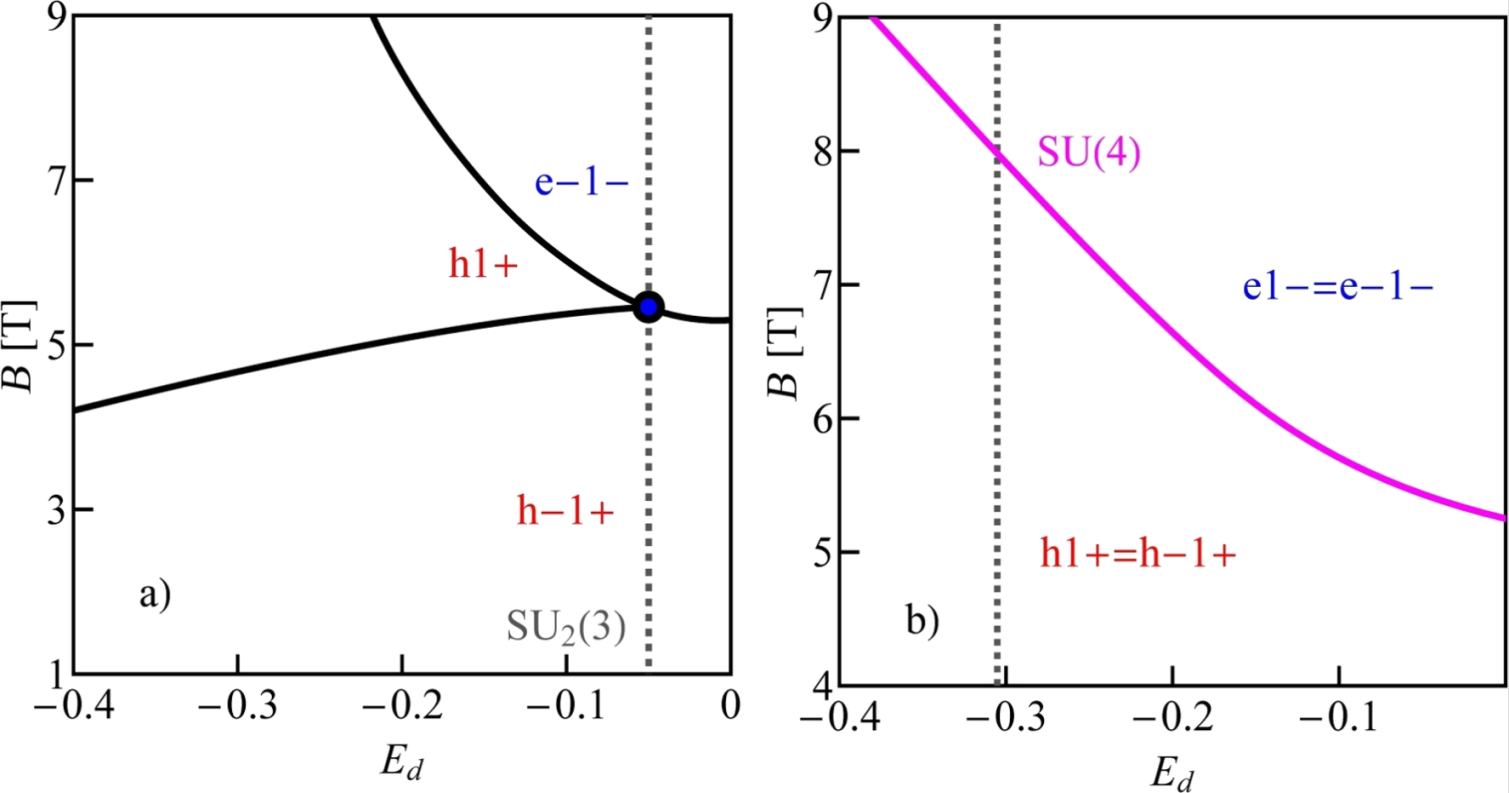
Electron–hole ground-state diagrams of CNTQD(33,30) for (a) θ = 86° and (b) θ = 90° (*U* = 6 meV, δ = 3/2 meV·nm, β = 37 meV·nm^2^).

Before discussing the correlation effects, let us show how the ground-state diagrams of an isolated dot change with the strength of SO interaction or with the orientation of the magnetic field. We restrict ourselves to the range of single occupation. Figure 9 presents ground-state diagrams for θ = 89° and several values of the SO coupling parameter. For δ = 3/2 meV·nm, in addition to the four double-degeneracy lines SU(2), also two SU(3) points are seen. These are SU_1_(3), where the two hole states degenerate with one electron state and the SU_2_(3) point, where two electron states degenerate with one hole state (Figure 9a). For δ = 0.9 meV·nm, four double-degeneracy lines (two lines of e–h degeneracy, one line of e–e, and one line of h–h degeneracy) meet in one point SU(4) (Figure 9b). Decreasing the SO interaction further to δ = 0.8 meV·nm results in the reappearance of the two SU(3) points again of similar character. However, now SU_1_(3) and SU_2_(3) change their relative position in the magnetic field/gate voltage plane (Figure 9c). For small values of SO interaction (e.g., δ = 0.4 meV·nm), no threefold-degeneracy point is observed (Figure 9d). Only a double electron–hole degeneracy line occurs for this strength of SO interaction. Figure 10 and Figure 9a illustrate modifications of the ground-state diagram with a change of the orientation of the magnetic field. For a transverse field, an electron–hole SU(4) line is visible (Figure 10b), for θ = 89°, two SU(3) points are observed in addition to the SU(2) lines (Figure 9a), and for a smaller angle of θ = 86°, only one single SU(3) point is left at the crossing of the double-degeneracy lines (Figure 10a).

**Figure 11:**
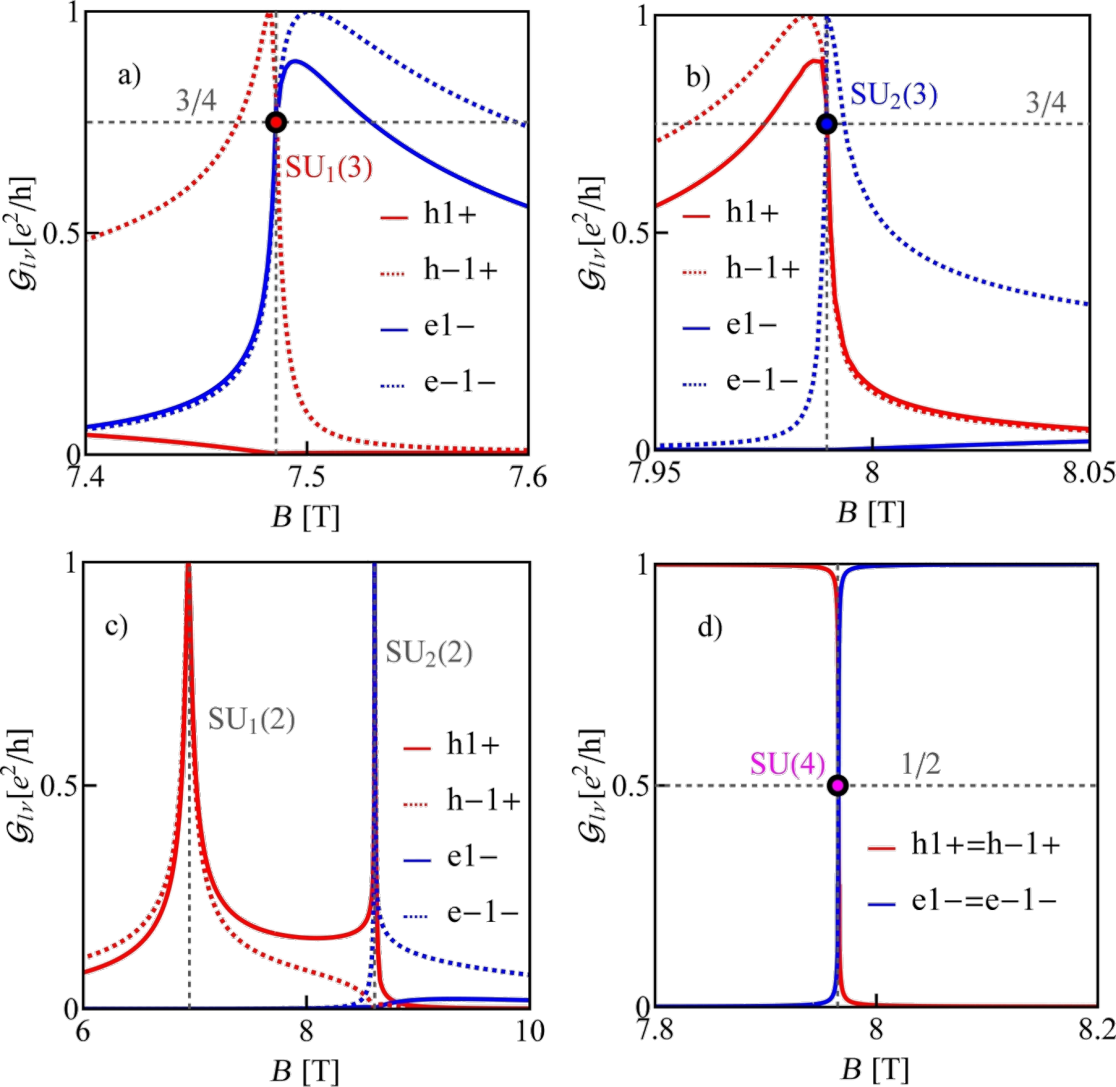
Partial conductances of CNTQD(33,30) in slanting magnetic fields for (a–c) θ = 89°, with curves plotted for cross sections from Figure 9a designated by the gray dotted lines, and for (d) θ = 90° for the cross sections marked by the dotted lines in Figure 9a (δ = 3/2 meV·nm, β = 37 meV·nm^2^, Γ = 0.01 meV and *U* = 6 meV).

In the case of strong coupling of the dot with the electrodes, the mentioned degeneracies of the electron and hole states enable the formation of Kondo resonances in which both electron and hole states participate. For a simpler numerical analysis, we restrict ourselves in our discussion to the subspace of only the two lowest electron states and the two highest hole states. This, as it is seen in Figure 8c and Figure 8d, is justified in the range of fields where the states degenerate because other states are distant on the energy scale. The considered regions of energies and fields are shown as grey boxes. The introduced restriction considerably simplifies the SBMFA calculations. Similarly to the cases discussed so far, many-body processes can be described by 16 slave-boson operators. Figure 11a–c present partial conductances of CNTQD(33,30) for a magnetic field directed at an angle of θ = 89° to the nanotube axis. According to the ground-state diagram presented earlier (Figure 9a) with double-degeneracy lines and two threefold-degeneracy points, one can expect Kondo SU(2) lines and two different Kondo SU(3) resonances. The vertical dashed lines in Figure 9a indicate the cross sections for which we present the conduction curves. Figure 11a presents the field dependence of the partial conductance along the cross section through the SU(3) Kondo state (SU_1_(3)) with fluctuating states |h−1+⟩, |e1−⟩, and |e−1−⟩. Figure 11b shows the field dependencies of the conductance through the SU(3) Kondo state (SU_2_(3)) involving the states |h1+⟩, |h−1+⟩, and |e−1−⟩. Figure 11c, in turn, presents the conductance for cross sections through two SU(2) points, namely the hole Kondo state SU_1_(2) (|h−1+⟩, |h1+⟩) and the electron–hole Kondo state SU_2_(2) (|h1+⟩, |e−1−⟩). At the SU(3) Kondo points, partial conductances corresponding to the states taking part in effective Kondo fluctuations reach a value of (3/4)(*e*^2^/*h*) and the contribution of the fourth channel is negligible. At the SU(2) points, two of the partial conductances take the unitary limit *e*^2^/*h*. Figure 11d shows partial conductances of the SU(4) Kondo effect occurring for transverse magnetic fields. They take a value of (1/2)(*e*^2^/*h*) each. Unlike the previously discussed SU(4) Kondo effect, the SU(4) Kondo resonance appears here for finite magnetic fields. The difference is that the states involved in the processes under discussion do not belong to the same shell anymore, as in the cases previously analyzed. Instead, two of them are electron states {|e−1−⟩, |e1−⟩} and two are hole states |h1+⟩, |h-1+⟩.

## Conclusion

We considered the effects of strong correlations in quantum dots formed in carbon nanotubes with small bandgap energies. These narrow bandgaps are formed in otherwise metallic nanotubes by curvature and can be modified by strain or twist. As a result of the non-linear dependence of the dot energies on the field, restoration of degeneracy is observed depending on the value of the atomic potential, controlled by the gate voltage, and on the strength of SO interactions. Lines of degeneracy occur in all Coulomb valleys. There are also threefold-degeneracy points in a finite field and fourfold-degeneracy points for zero magnetic field. The resonances of the spin SU(2) Kondo effect are characterized by a non-zero orbital moment (quenched spin magnetic moment) and orbital Kondo resonances exhibiting non-zero spin magnetic moment. Kondo SU(3) resonances have non-zero orbital and spin moments, and in the Kondo SU(4) state both moments are quenched. By changing the value of the bandgap energy by stress, one can move high-symmetry points between different Coulomb valleys. The SU(4) point occurs for zero field. If it appears in the double-occupied region, it separates the SU(3) lines such that in different parts of the line there are different SU(3) Kondo resonances associated with other sets of the dot states. In a quantum dot formed in a narrow-bandgap nanotube, the electron and hole levels are energetically sufficiently close that some of them can degenerate in a magnetic field. This opens the possibility of Kondo effects of various symmetries in which both electron and hole states participate. SU(3) points appear for fields close to a perpendicular orientation of the field with respect to the nanotube axis, and an electron–hole SU(4) Kondo effect is induced in a perpendicular field.
